# Environmental contamination with *Toxocara* eggs: a quantitative approach to estimate the relative contributions of dogs, cats and foxes, and to assess the efficacy of advised interventions in dogs

**DOI:** 10.1186/s13071-015-1009-9

**Published:** 2015-07-28

**Authors:** Rolf Nijsse, Lapo Mughini-Gras, Jaap A. Wagenaar, Frits Franssen, Harm W. Ploeger

**Affiliations:** Department of Infectious Diseases and Immunology, Faculty of Veterinary Medicine, Utrecht University, P.O. box 80.165, 3508 TD Utrecht, The Netherlands; National Institute for Public Health and the Environment, Centre for Infectious Disease Control, P.O. box 1, Bilthoven, 3720 BA The Netherlands; Central Veterinary Institute of Wageningen UR, Houtribweg 39, 8221 RA Lelystad, The Netherlands

**Keywords:** *Toxocara*, Eggs, Dogs, Cats, Foxes, Contribution, Contamination, Environment, Deworming, Clean-up

## Abstract

**Background:**

Environmental contamination with *Toxocara* eggs is considered the main source of human toxocariasis. The contribution of different groups of hosts to this contamination is largely unknown. Current deworming advices focus mainly on dogs. However, controversy exists about blind deworming regimens for >6-month-old dogs, as most of them do not actually shed *Toxocara* eggs. We aim to estimate the contribution of different non-juvenile hosts to the environmental *Toxocara* egg contamination and to assess the effects of different *Toxocara*-reducing interventions for dogs.

**Methods:**

A stochastic model was developed to quantify the relative contribution to the environmental contamination with *Toxocara* eggs of household dogs, household cats, stray cats, and foxes, all older than 6 months in areas with varying urbanization degrees. The model was built upon an existing model developed by Morgan et al. (2013). We used both original and published data on host density, prevalence and intensity of infection, coprophagic behaviour, faeces disposal by owners, and cats’ outdoor access. Scenario analyses were performed to assess the expected reduction in dogs’ egg output according to different deworming regimens and faeces clean-up compliances. Estimates referred to the Netherlands, a country free of stray dogs.

**Results:**

Household dogs accounted for 39 % of the overall egg output of >6-month-old hosts in the Netherlands, followed by stray cats (27 %), household cats (19 %), and foxes (15 %). In urban areas, egg output was dominated by stray cats (81 %). Intervention scenarios revealed that only with a high compliance (90 %) to the four times a year deworming advice, dogs’ contribution would drop from 39 to 28 %. Alternatively, when 50 % of owners would always remove their dogs’ faeces, dogs’ contribution would drop to 20 %.

**Conclusion:**

Among final hosts of *Toxocara* older than 6 months, dogs are the main contributors to the environmental egg contamination, though cats in total (i.e. both owned and stray) transcend this contribution. A higher than expected compliance to deworming advice is necessary to reduce dogs’ egg output meaningfully. Actions focusing solely on household dogs and cats are unlikely to sufficiently reduce environmental contamination with eggs, as stray cats and foxes are also important contributors.

## Background

Ocular and visceral larva migrans, as well as exacerbation of asthmatic allergies, are often associated with *Toxocara* spp. infection in humans [1–3]. This is supported by evidence from serological studies [2], although conclusive diagnosis can be very difficult [4] and seroconversion occurs often in people without recognized clinical symptoms [5].

Environmental contamination with *Toxocara* eggs is believed to be the main source of human infections, which are usually caused by accidental ingestion of infective eggs present in the environment. Of the different *Toxocara* species, *Toxocara canis* and *Toxocara cati* are considered to pose the highest zoonotic risk. Although there are incidental reports of *Toxocara vitulorum* [6], this species is not thought to be of significant epidemiological importance for human toxocariasis in the Netherlands. Therefore, in order to reduce the environmental contamination with *Toxocara* eggs, one should focus on the main egg shedders of *T. canis* and *T. cati*, i.e. dogs, cats, or foxes. Of these, dogs are probably the population of hosts in which *Toxocara* infections can be controlled the best by the owners, because, in contrast to cats, there is no notable population of stray dogs in the Netherlands.

The actual contribution of household dogs to the environmental contamination with *Toxocara* eggs is largely unknown, and so are the contributions of foxes and (either owned or un-owned) cats, which are commonly present in the Netherlands. A model quantifying the relative contributions of different final hosts to the environmental contamination with *Toxocara* eggs in the city of Bristol, UK [7], revealed that dogs, especially those in the age group of <12 weeks, were responsible for most of the total *Toxocara* egg output, even if it was assumed that 75 % of the produced eggs did not reach the environment directly due to confinement of dogs at such a young age. Morgan et al. [7] further showed by simulation that the proportion of *T. canis* eggs reaching the environment is, not surprisingly, strongly dependent on the rates of removal of dog faeces by owners, but actual data about the compliance of dog owners to clean-up their dogs’ faeces was not available and therefore could not be incorporated in the model. What also could not be considered in that model was the level of outdoor access of household cats, and the frequency of preferred use of the litterbox, or that foxes may have more or less access to some areas depending on their degree of urbanization. Accounting for the degree of access to different (outdoor) areas and removal of faeces is therefore likely to provide novel insights in the relative contributions of different hosts older than 6 months (hereafter referred to as non-juvenile hosts) to the environmental contamination by *Toxocara* eggs.

Currently, the European Scientific Counsel Companion Animal Parasites (ESCCAP) recommends to deworm adult dogs (>6 months of age) at least four times a year [8] to reduce the impact of patent infections on the environmental contamination with *Toxocara* eggs. However, this recommendation is not well supported by evidence and, as it is voluntary, it leaves ample room for dog owners to deworm their dogs (or not) in whatever frequency they like. As it cannot be expected that owners make these decisions based on adequate knowledge of the public health issues related to patent *Toxocara* infections [9], modelling the expected outcome of differing deworming frequencies might help determine the extent to which efforts should be put into convincing dog owners to comply with recommended treatment strategies. Because final hosts younger than 6 months of age are unlikely to have acquired age resistance against patent infections with *Toxocara* spp*.*, they are believed to contribute by far the most to the overall *Toxocara* egg production [7, 10–12]. Accordingly, the current deworming advice for these young animals, which is based on the prepatent periods of intra-uterine and lactogenic infection, as well as infection by ingesting embryonated eggs, should be propagated and enforced. This means that puppies are to be dewormed every 2 weeks up to the age of 8 weeks, followed by monthly deworming up to the age of 6 months. The same applies to the advice of daily clean-up and disposal of their faeces by the owners. This advice is to be communicated to owners of puppies and kittens without reservation. There is, however, controversy about the necessity of the advocated deworming regimen for dogs older than 6 months, as the majority of household dogs (>90 %) does not actually shed *Toxocara* eggs [9, 13–15]. Additionally, for dogs older than 6 months, a mean prepatent period to serve as a guideline for deworming individual dogs cannot be as easily defined as in puppies. Puppies will not yet have developed an age resistance. Age resistance leads to mostly somatic instead of tracheal migration of larvae hatched from infective eggs. Therefore, when dogs have built up an age resistance, infection with embryonated eggs will not usually lead to a patent infection. Instead of migrating through the lungs, larvae cumulate in the somatic tissues which results in a prolonged and unpredictable prepatent period. For this reason, the present study focussed on animals older than 6 months, for which the propagated deworming advice is arguable.

Building upon the work of Morgan et al. [7], the main aim of this study was to develop a quantitative modelling approach to estimate stochastically the relative contributions of different non-juvenile host species to the environmental contamination with *Toxocara* eggs. Not only the host density, prevalence and intensity of infection, but also the degree of access to different (outdoor) areas and removal of faeces were taken into account. A comprehensive data set was then compiled using both published and original data to quantify the relative contributions to the overall *Toxocara* egg output in the Netherlands of non-juvenile household dogs, foxes, owned and un-owned cats (hereafter referred to as stray cats), all older than 6 months. Another aim of this study was to assess the effects of implementing different deworming regimens and compliance to faeces clean-up policies for household dogs on the total environmental contamination with *Toxocara* eggs.

## Methods

### Modelling approach

Our modelling approach builds upon an existing model [7] to quantify the number of *Toxocara* eggs released into the environment by non-juvenile (≥6 month-old) final hosts (dogs, household cats, stray cats, and foxes) in the Netherlands. As there are virtually no stray dogs in the Netherlands [15], only the contribution of household dogs to the environmental contamination with *Toxocara* eggs was quantified. Conversely, both stray and household cats were considered.

The computational method used to estimate the overall daily egg output of non-juvenile dogs, household cats, stray cats and foxes (hereafter referred to interchangeably as hosts) in the Netherlands was the same for each of these hosts, with some adaptations depending on the data available and biological characteristics of the host in question (see section Description of the model). Since degree of urbanization and age are major determinants of host population size and frequency of egg shedding hosts [7, 9, 12, 16, 17], the degree of urbanization and the age structure were expected to have a strong effect on the estimates. Therefore, for all hosts, the daily egg output was estimated separately for young adults (6–12 months of age) and adults (>12 months of age), and for urban (>2500 addresses/km^2^), intermediate (500–2500 addresses/km^2^) and rural (<500 addresses/km^2^) areas. The age categorization was based on a previous study [9] reporting a significantly higher risk of shedding *Toxocara* eggs in 6–12 month-old dogs compared to older age groups. The degree of urbanization, expressed in addresses/km^2^ at the postal code area level, was based on the official categorization of the Dutch Central Bureau of Statistics used in other studies in the Netherlands, e.g. [18, 19].

### Description of the model

Let *i* denote the host, with *i* = 1 (dogs), 2 (household cats), 3 (stray cats), and 4 (foxes); let *j* denote the age group which individuals of host *i* belong to, with *j* = 1 (young adults) and 2 (adults); and let *z* denote the urbanization degree of the postal code area where individuals of host *i* and age group *j* live in, with *z* = 1 (urban areas), 2 (intermediate areas), and 3 (rural areas). The expected number of *Toxocara* eggs per km^2^ released each day into the environment by host *i* of age group *j* living in area *z*, denoted as *E*_*ijz*_, is estimated as:

$$ {E}_{ijz}\sim Poisson\left({\lambda}_{ijz}\right) $$

$$ {\lambda}_{ijz}={D}_{ijz}\times {P}_{ijz}\times {F}_i\times {I}_{ij} $$

where *D*_*ijz*_ is the overall density (individuals/km^2^) of host *i* and age group *j* living in area *z*; *P*_*ijz*_ is the true prevalence of patent *Toxocara* infections among individuals of host *i* and age group *j* living in area *z*; *F*_*i*_ is the average daily faecal output (grams of faeces per individual per day) of host *i* released into the environment; and *I*_*ij*_ is the average intensity of infection, expressed as eggs per gram of faeces (EPG), in host *i* and age group *j*. Full details on the estimation and data sources of these parameters are reported in Table 1. A sum of the egg outputs over age groups and areas, weighted by the size of the areas themselves (*a*_*z*_, expressed in km^2^), gives the overall daily egg output of host *i* in the Netherlands, denoted by:

$$ {E}_i={\displaystyle {\sum}_j{\displaystyle {\sum}_z{E}_{ijz}}}\times {a}_z $$

The model was based on a Monte Carlo simulation implemented in @Risk (Palisade Corp., USA) by setting 10,000 iterations with the Latin hypercube sampling technique and a seed of one. Model convergence was monitored to check how statistics changed on the output distributions. Convergence testing was enabled every 100 iterations. Default convergence options were used, with a convergence tolerance of 3 % and a confidence interval of 95 %; all models showed optimal convergence.

**Table 1:** Model parameters and sources, as used in the model. Parameter means are shown in Table 3

Parameter	Description	Estimation	Source
Dogs			
*D*_1*jz*_	Density of dogs of age group *j* in area *z*	Data	[20]
*P*_1*jz*_	Prevalence of *Toxocara* patent infection in dogs of age group *j* in area *z*	= *p*_1*jz*_ × *c*_1*jz*_	See below
*p*_1*jz*_	Coprological prevalence of *Toxocara* egg shedding dogs of age group *j* in area *z*	~Beta (*a*_1*jz*_ + 1, *b*_1*jz*_ + 1), where: *a*_1,1,1_ = 2, *b*_1,1,1_ = 47; *a*_1,2,1_ = 5, *b*_1,2,1_ = 129; *a*_1,1,2_ = 10, *b*_1,1,2_ = 122; *a*_1,2,2_ = 12, *b*_1,2,2_ = 389; *a*_1,1,3_ = 6, *b*_1,1,3_ = 43; *a*_1,2,3_ = 7, *b*_1,2,3_ = 137	[9]
*c*_1*jz*_	Proportion of dogs of age group *j* in area *z* that do not display a coprophagic behaviour	~Beta (*a*_1*jz*_ + 1, *b*_1*jz*_ + 1), where: *a*_1,1,1_ = 26, *b*_1,1,1_ = 22; *a*_1,2,1_ = 80, *b*_1,2,1_ = 54; *a*_1,1,2_ = 56, *b*_1,1,2_ = 75; *a*_1,2,2_ = 226, *b*_1,2,2_ = 175; *a*_1,1,3_ = 29, *b*_1,1,3_ = 18; *a*_1,2,3_ = 89, *b*_1,2,3_ = 55	[9]
*F*_1*jz*_	Average faecal output of a dog of age group *j* released daily into the environment of area *z*	= *f*_1_ × *s*_1*jz*_	See below
*f*_1_	Average faecal output of a dog	~Pert (21, 254, 1074)	[22–33]
*s*_1*jz*_	Proportion of dog owners that do not comply to dog waste clean-up policies for dogs of age group *j* in area *z*	~Beta (*a*_1*jz*_ + 1, *b*_1*jz*_ + 1), where: *a*_1,1,1_ = 20, *b*_1,1,1_ = 28; *a*_1,2,1_ = 80, *b*_1,2,1_ = 54; *a*_1,1,2_ = 87, *b*_1,1,2_ = 44; *a*_1,2,2_ = 277, *b*_1,2,2_ = 154; *a*_1,1,3_ = 27, *b*_1,1,3_ = 20; *a*_1,2,3_ = 106, *b*_1,2,3_ = 37	[9]
*I*_1*j*_	Infection intensity (EPG) for dogs of age group *j*	*I*_1*,*1_ ~ Poisson (341.2); *I*_1*,*2_ ~ Poisson (163.7)	[34]
Household cats			
*D*_2*jz*_	Density of household cats of age group *j* in area *z*	Data	[20]
*P*_2*jz*_	Prevalence of *Toxocara* patent infection in household cats of age group *j* in area *z*	~Beta (*a*_2*jz*_ + 1, *b*_2*jz*_ + 1), where: *a*_2,1,1_ = 0, *b*_2,1,1_ = 2; *a*_2,2,1_ = 0, *b*_2,2,1_ = 18; *a*_2,1,2_ = 2, *b*_2,1,2_ = 15; *a*_2,2,2_ = 8, *b*_2,2,2_ = 52; *a*_2,1,3_ = 2, *b*_2,1,3_ = 1; *a*_2,2,3_ = 5, *b*_2,2,3_ = 12	[Nijsse, unpublished data]
*F*_2*jz*_	Average faecal output of a household cat of age group *j* released daily into the environment of area *z*	= *f*_2_ × *o*_2*jz*_	See below
*f*_2_	Average faecal output of a household cat	~Pert (10.2, 19.4, 52.4)	[35–39]
*o*_2*jz*_	Proportion of household cats of group *j* in area *z* with outdoor access	~Beta (*a*_2*jz*_ + 1, *b*_2*jz*_ + 1), where: *a*_2,1,1_ = 1, *b*_2,1,1_ = 1; *a*_2,2,1_ = 5, *b*_2,2,1_ = 13; *a*_2,1,2_ = 3, *b*_2,1,2_ = 13; *a*_2,2,2_ = 45, *b*_2,2,2_ = 13; *a*_2,1,3_ = 2, *b*_2,1,3_ = 1; *a*_2,2,3_ = 14, *b*_2,2,3_ = 3	[Nijsse, unpublished data]
*I*_2*j*_	Infection intensity (EPG) for household cats of age group *j*	*I*_2*,*1_ ~ Poisson (372.8); *I*_2*,*2_ ~ Poisson (81.7)	[40]
Stray cats			
*D*_3*jz*_	Density of stray cats of age group *j* in area *z*	~Pert (135,000,667,500,1,200,000) × *D*_2*jz*_ /(∑_*j*_ ∑_*i*_ *D*_2*jz*_)	Personal communication: preliminary estimate of feral cat project WUR Wageningen
*P*_3*j*_	Prevalence of *Toxocara* patent infection in stray cats of age group *j*	~Beta (*a*_3*jz*_ + 1, *b*_3*jz*_ + 1), where: *a*_3,1,1_ = 16, *b*_3,1,1_ = 12; *a*_3,2,1_ = 17, *b*_3,2,1_ = 8; *a*_3,1,2_ = 16, *b*_3,1,2_ = 12; *a*_3,2,2_ = 17, *b*_3,2,2_ = 8; *a*_3,1,3_ = 16, *b*_3,1,3_ = 12; *a*_3,2,3_ = 17, *b*_3,2,3_ = 8	[11]
*F*_3_	Average faecal output of a stray cat	~Pert (10.2, 19.4, 52.4)	[35–39]
*I*_3*j*_	Infection intensity (EPG) for stray cats of age group *j*	*I*_3*,*1_ ~ Poisson (372.8); *I*_3*,*2_ ~ Poisson (81.7)	[40]
Foxes			
*D*_4*jz*_	Density of foxes of age group *j* in area *z*	~Pert (0.5, 2.25, 4) × *d*_4*jz*_/(∑_*j*_∑_*i*_ *d*_4*jz*_)	[41]
*d*_4*jz*_	Total number of foxes of age group *j* shot in area *z*	Data	[41]
*P*_4*jz*_	Prevalence of *Toxocara* patent infection in foxes of age group *j* in area *z*	~Beta (*a*_4*jz*_ + 1, *b*_4*jz*_ + 1), where: *a*_4,1,1_ = 1, *b*_4,1,1_ = 1; *a*_4,2,1_ = 1, *b*_4,2,1_ = 1; *a*_4,1,2_ = 18, *b*_4,1,2_ = 28; *a*_4,2,2_ = 9, *b*_4,2,2_ = 12; *a*_4,1,3_ = 57, *b*_4,1,3_ = 74; *a*_4,2,3_ = 19, *b*_4,2,3_ = 39	[41]
*F*_4_	Average faecal output of a fox	Log(*F*_4_) ~ Normal (95, 18)	[42]
*I*_4*j*_	Infection intensity (EPG) for foxes of age group *j*	*I*_4*,*1_ ~ Poisson (157); *I*_4*,*2_ ~ Poisson (366)	[12]

Description, estimation and data sources of the model parameters used to quantify the number of *Toxocara* eggs released into the environment by non-juvenile (≥6 month-old) dogs, household cats, stray cats and foxes in the Netherlands. Parameter means are shown in Table 3

### Data sources and model parameterization

#### Dogs

The density of dogs by age group and urbanization degree (*D*_1*jz*_) was obtained from a study on the pet population in the Netherlands in 2011 included in a report compiled by the University of Applied Sciences of Den Bosch and the Council of Animal Affairs in the Hague, the Netherlands, under the mandate of the Dutch Ministry of Economic Affairs, Agriculture and Innovation [20]. *Toxocara* egg prevalence in dog faeces by age group and urbanization degree (*p*_1*jz*_) was obtained from a large study on the prevalence, risk factors and owners’ attitude towards deworming for *Toxocara* based on 916 dogs of ≥6 months of age that was conducted in the Netherlands between July 2011 and August 2012 [9]. Dog owners voluntarily participated in this study and agreed on publication of the anonymised data. Such prevalence was adjusted for the likelihood for these dogs to display coprophagic behaviour, as this causes overestimation of the true prevalence due to the passive passage of helminth eggs through the dog’s digestive tract following ingestion of “egg-contaminated” faeces [21]. Coprophagy-adjusted *Toxocara* egg prevalence in dog faeces was estimated as *P*_1*jz*_ = *p*_1*jz*_ × *c*_1*jz*_, where *p*_1*jz*_ is the observed coprological prevalence of *Toxocara* eggs in dogs of age group *j* living in area *z*, and *c*_1*jz*_ is the corresponding age-and area-specific proportion of dogs that do not display a coprophagic behaviour as provided by Nijsse et al. [9]. Both *p*_1*jz*_ and *c*_1*jz*_ parameters were modelled as Beta distributions (see Table 1).

The average faecal output of a (Dutch) dog, denoted as *f*_1_, was derived by calculating the pooled, sample size-weighted mean faecal output (expressed as grams of faeces per kilogram of dog’s live body weight), over 12 different studies on dog food digestibility [22–33], weighted by the average bodyweight of a Dutch dog being 21.5 kg [20]. Minimum and maximum faecal outputs were derived proportionally by taking the Chihuahua and the Great Dane as reference breeds for the extremes of the dog faecal output range so that *f*_1_ could be modelled as a Pert distribution (Table 1). Dog faecal output was adjusted for age-and area-specific likelihood for dog faeces to be cleaned-up by their owners as to estimate the amount of dog faeces that is actually released into the environment (*F*_1_). This was estimated as *F*_1(*jz*)_ = *f*_1_× *s*_1*jz*_, where *f*_1_ is the above mentioned average faecal output of a (Dutch) dog and *s*_1*jz*_ is the proportion of dog owners that does not comply to dog waste clean-up policies among those owning dogs of age group *j* living in area *z*. Parameter *s*_1*jz*_ was modelled as Beta distribution (Table 1) for which priors were obtained from Nijsse et al. [9].

Infection intensity (EPG) of *Toxocara* in dogs by age group (*I*_1*j*_) was obtained from Sowemimo [34] and modelled as a Poisson distribution (Table 1). This parameter did not change over degrees of urbanization, but only over age groups, as it was assumed to be a parasite-related property in a given host, irrespective of the area that host lives in.

#### Household cats

The density of household cats by age group and urbanization degree (*D*_2*jz*_) was obtained from the same source as dogs [20]. *Toxocara* prevalence in household cats by age group and urbanization degree (*P*_2*jz*_) was obtained from a coprological study comprising126 owned cats in the Netherlands conducted at the Faculty of Veterinary Medicine of Utrecht University between October 2011 and February 2012 (Nijsse, unpublished data). Prevalence was modelled as Beta distribution (Table 1). All cat owners voluntarily participated in this study and agreed on publication of the anonymised data.

Similar to dogs, the average faecal output of a cat, denoted as *f*_2_, was derived by calculating the pooled, sample size-weighted mean faecal output (grams of faeces per kilogram of cat’s live body weight), over five different studies on cat food digestibility [35–39]. Minimum and maximum faecal outputs were derived proportionally by taking the Singapura and the Maine Coon as reference breeds for the extremes of the cat faecal output range so that *f*_2_ could be modelled as a Pert distribution (Table 1). Faecal output of household cats was adjusted for the age-and area-specific likelihood for household cat faeces to be actually released into the environment because these cats have access to outdoor areas. This was estimated as *F*_2(*jz*)_ = *f*_2_× *o*_2*jz*_, where *f*_2_ is the above mentioned average faecal output of a cat and *o*_2*jz*_ is the proportion of household cats of age group *j* in area *z* having outdoor access. Parameter *o*_2*jz*_was modelled as Beta distribution (Table 1) for which priors were obtained from the results of the above mentioned study (Nijsse, unpublished data).

Similar to dogs, EPG in household cats by age group (*I*_2*j*_) was obtained from Sowemimo [40] and modelled as a Poisson distribution (Table 1), with no changes over degrees of urbanization.

#### Stray cats

There were no precise data on the density of stray cats by age group and urbanization degree in the Netherlands (*D*_3*jz*_). At the time of writing, a survey to determine the number of stray cats in the Netherlands was ongoing at Wageningen University (http://www.wageningenur.nl/nl/project/Nederlandse-zwerfkatten-in-beeld.htm). They provided us with the most likely estimate of the stray cat population in the Netherlands based on their preliminary data. This estimate is between 135,000 and 1,200,000 stray cats. Using these priors, a Pert distribution was used to estimate the total stray cat population in the Netherlands, which was distributed over age groups and urbanization degrees based on the observed age structure and urban-to-rural gradient of household cats (Table 1). Inherent to this approach is the assumption that the stray cat population follows that of household cats in terms of both age composition and spatial distribution.

*Toxocara* prevalence in stray cats by age group (*P*_3*j*_) was obtained from O’Lorcain [11] and modelled as Beta distribution (Table 1). Because of the lack of data, this parameter could not vary over degrees of urbanization, but only over age groups. The average faecal output of a stray cat was the same as that of household cats (Description of the model), but it was not adjusted for outdoor access since by definition all stray cats live outside and all their faeces is released into the environment. EPG in stray cats by age group (*I*_3*j*_) was the same as that of household cats (Table 1).

#### Foxes

There were no precise data on the density of foxes by age group and urbanization degree in the Netherlands (*D*_4*jz*_). Franssen et al. [41] estimated an overall density of 0.5 to 4.0 foxes per km^2^ in the Netherlands. Using these priors, a Pert distribution was used to estimate the average fox density in the Netherlands. This was then distributed over age groups and urbanization degrees based on the age structure and urban-to-rural gradient observed in a sample of 288 shot foxes submitted by hunters for routine inspection to the Dutch National Institute for Public Health and Environment between October 2010 and April 2012 [41] (Table 1). *Toxocara* prevalence in foxes by age group and urbanization degree (*P*_4*jz*_) was also obtained from Franssen et al. [41], who examined the intestine of a subset of 262 foxes for the recovery of adult worms. Prevalence was modelled as a Beta distribution (Table 1). The mean and standard deviation of the faecal output of foxes were provided by Nissen et al. [42] so that the fox faecal output (*F*_4_) could be modelled as a log normal distribution (Table 1). EPG in foxes by age group (*I*_4*j*_) was obtained from Saeed et al. [12] and modelled as a Poisson distribution (Table 1), with no changes over degrees of urbanization.

### Scenario analysis

Since dogs are the traditional target of control activities for *Toxocara* infection, different scenarios were simulated to quantify the impact of varying deworming regimens for dogs on the daily egg output of dogs in the Netherlands. These scenarios were run in parallel with those assessing the sole effect of removal of dog faeces. 16 scenarios were simulated in which four putatively advised deworming regimens (i.e. twice a year, four times a year, six times a year, and 12 times a year) were applied. For this simulation the use of short-acting deworming compounds is assumed at four different rates of compliance (i.e. 30, 50, 70 and 90 %), with an average prepatent period of 30 days [43, 44] and full efficacy of the deworming treatment. Since our model was based on real-world data, of which a subset was already used by Nijsse et al. [9], these scenarios were simulated on top of a background of observed deworming regimens and respective compliance rates present in the Dutch dog population (i.e. twice a year: 21.0 % of dogs; four times a year: 17.5 % of dogs; six and 12 times a year: unknown). Another four scenarios were simulated in which the observed compliance rates to dog waste clean-up policies (see Table 2) were increased by 20, 50, 70 and 90 %.

**Table 2:** Estimated percentages of coprophagic behaviour, clean-up behavior of owners and outdoor access of household cats

Area	Age group	Coprophagic dogs (*c*_1_), %	percentage of dog owners that never/rarely clean up feces (*s*_1_), %	Household cats with outdoor access (*o*_2_), %
Urban	Young adults	54.00 (40.23–67.46)	42.00 (28.81–55.78)	50.00 (9.41–90.56)
Urban	Adults	59.56 (51.22–67.62)	59.56 (51.22–67.63)	30.00 (12.57–51.20)
Intermediate	Young adults	42.86 (34.59–51–32)	66.17 (57.93–73.93)	22.22 (6.80–43.41)
Intermediate	Adults	56.33 (51.46–61.13)	64.20 (59.63–68.65)	76.67 (65.26–86.38)
Rural	Young adults	61.22 (47.34–74.23)	57.14 (43.21–70.51)	60.00 (19.39–93.24)
Rural	Adults	61.64 (53.64–69.34)	73.79 (66.36–80.60)	78.95 (58.56–93.59)

Estimated mean and 95 % confidence interval of the posterior distribution of the rates of dogs displaying coprophagic behaviour, percentage of dog owners that never/rarely clean up feces, and outdoor access of household cats for young adults (6–12 month-old) and adults (>12 month-old) in urban, intermediate and rural areas in the Netherlands

## Results

An estimated 84,100 (95 % CI: 55,200–120,500) *Toxocara* eggs per km^2^ per day are shed, on average, by non-juvenile hosts (>6 months) in the Netherlands. This corresponded to an average egg output of 1.46 × 10^6^ (0.63 × 10^6^–2.76 × 10^6^) eggs per km^2^ per day in urban areas, 109,500 (54,500–196,600) eggs per km^2^ per day in intermediate areas, and 38,200 (21,200–61,700) eggs per km^2^ per day in rural areas.

### Estimated host contributions to environmental egg contamination

**Fig. 1:**
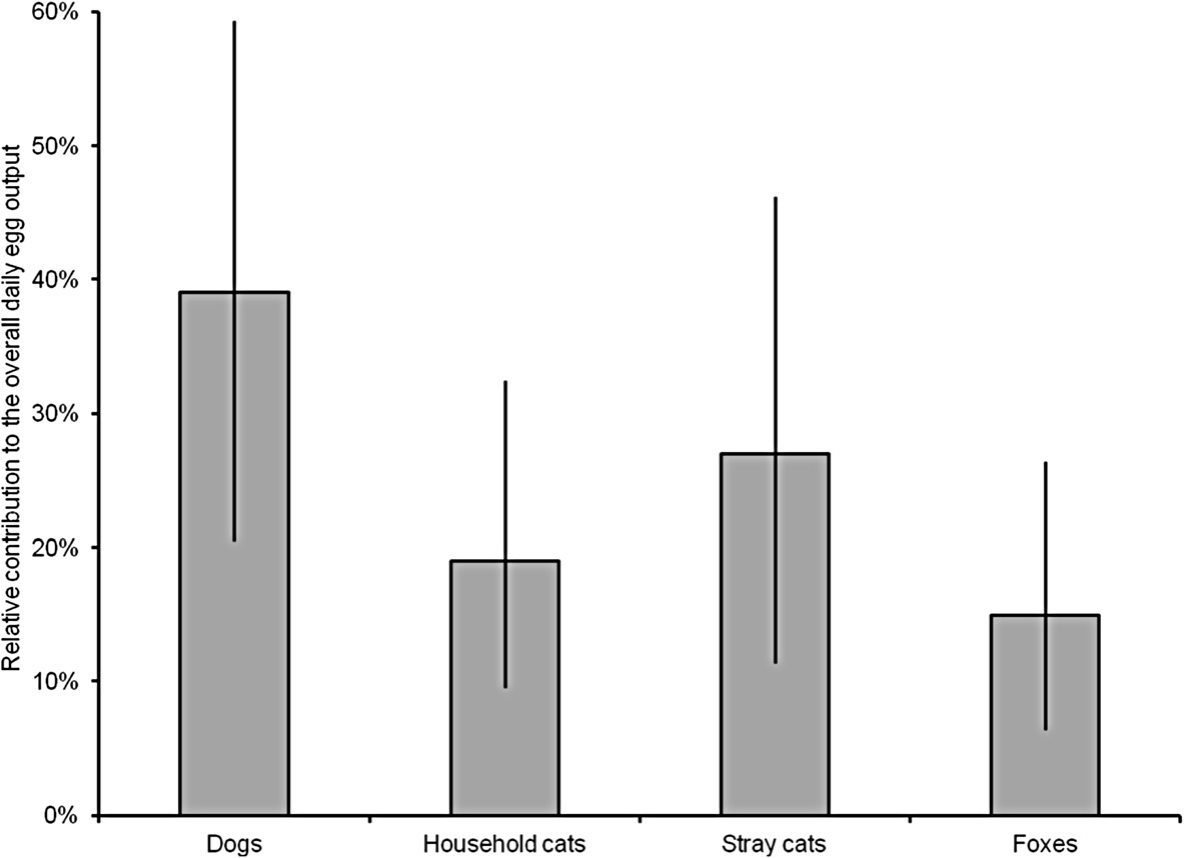
Relative contributions to environmental contamination with *Toxocara* eggs in the whole of the Netherlands. Estimated relative contributions (%) of non-juvenile (≥6 month-old) dogs, household cats, stray cats, and foxes to the environmental contamination with *Toxocara* eggs in the whole of the Netherlands. Error bars represent 95 % confidence intervals

Of the four putative non-juvenile hosts groups considered (dogs, household cats, stray cats, and foxes), dogs were estimated to be the most important contributor to the environmental contamination with *Toxocara* eggs (Fig. 1). They accounted for 39.1 % of the overall daily egg output of non-juvenile hosts in the Netherlands, followed by stray cats (27.0 %), household cats (19.0 %), and foxes (14.9 %). This was in spite of the relatively low prevalence of patent *Toxocara* infections in dogs, but by virtue of their high population density and faecal output (Table 3), as well as low compliance of dog owners to dog waste clean-up policies (Table 2). However, when summing the contributions of household and stray cats together (46.0 %), it appeared that non-juvenile cats as a whole are the primary contributor among the considered host groups. The relatively large population size and high prevalence of egg-shedding cats, either owned or stray (Table 3), along with a high proportion of household cats with outdoor access (Table 2), meant that non-juvenile cats were estimated to be the most important source of *Toxocara* eggs in the Netherlands, despite their relatively low faecal output and intensity of infection (Table 3).

**Table 3:** Estimated mean (with 95 % confidence intervals) of the posterior distributions of model parameters

	Urban areas		Intermediate areas		Rural areas	
	Young adults	Adults	Young adults	Adults	Young adults	Adults
Population density (*D*), heads/km^2^						
Dogs^a^	9	208.6	3.4	79.7	0.4	8.7
Household cats^a^	32.5	755.5	5.7	131.8	0.5	12.5
Stray cats	34.8 (15.1–54.4)	808.0 (352.7–1263.8)	0.3 (0.1–0.5)	6.9 (3.0–10.9)	0.01 (0.006–0.02)	0.3 (0.1–0.5)
Foxes	0.004 (0.002–0.006)	0.005 (0.002–0.007)	0.3 (0.1–0.4)	0.3 (0.2–0.5)	0.7 (0.3–1.1)	0.9 (0.4–1.4)
Prevalence (*P*), %						
Dogs^d^	3.2 (0.7–7.6)	2.6 (1.0–5.1)	3.5 (1.7–5.9)	1.8 (1.0–2.9)	8.4 (3.4–15.3)	3.4 (1.5–6.0)
Household cats	25.0 (0.8–70.8)	5.0 (0.1–17.6)	15.8 (3.6–34.7)	14.52 (7.0–24.2)	60.0 (19.4–93.2)	31.6 (13.3–53.5)
Stray cats^c^	56.7 (38.9–73.6)	66.7 (48.2–82.8)	56.7 (38.9–73.6)	66.7 (48.2–82.8)	56.7 (38.9–73.5)	66.7 (48.2–82.8)
Foxes^b^	50.0 (9.4–90.6)	50.0 (9.4–90.6)	39.6 (26.4–53.6)	43.5 (24.4–63.6)	43.6 (35.3–52.1)	33.3 (22.1–45.6)
Faecal output (*F*), g/day						
Dogs^e^	147.7 (27.8–332.6)	209.6 (40.5–452.3)	232.9 (44.6–504.8)	225.9 (43.4–487.0)	201.1 (38.2–447.7)	259.6 (49.9–559.3)
Household cats^f^	11.7 (1.9–27.0)	7.0 (2.3–14.9)	5.2 (1.3–12.2)	17.9 (9.0–30.8)	14.0 (3.7–29.4)	18.5 (9.0–32.4)
Stray cats^g^	23.4 (12.1–39.5)	23.4 (12.1–39.5)	23.4 (12.1–39.5)	23.4 (12.1–39.5)	23.4 (12.1–39.5)	23.4 (12.1–39.5)
Foxes^g^	95.0 (64.6–134.9)	95.0 (64.6–134.9)	95.0 (64.6–134.9)	95.0 (64.6–134.8)	95.0 (64.6–134.9)	95.0 (64.6–134.9)
Infection intensity (*I*), eggs/g faeces						
Dogs^h^	341.2 (305–378)	163.7 (139–189)	341.2 (305–378)	163.7 (139–189)	341.2 (305–378)	163.7 (139–189)
Household cats^h^	372.8 (335–411)	81.7 (64–100)	372.8 (335–411)	81.7 (64–100)	372.8 (335–411)	81.7 (64–100)
Stray cats^h^	372.8 (335–441)	81.7 (64–100)	372.8 (335–441)	81.7 (64–100)	372.8 (335–441)	81.7 (64–100)
Foxes^h^	157.0 (133–182)	366.0 (329–404)	157.0 (133–182)	366.0 (329–404)	157.0 (133–182)	366.0 (329–404)

Estimated mean and 95 % confidence intervals of the posterior distribution of the host population density, prevalence of patent *Toxocara* infection, average daily faecal output released into the environment, and infection intensity for young adult (6–12 month-old) and adult (>12 month-old) dogs, household cats, stray cats and foxes in urban, intermediate and rural areas in the Netherlands

^a^Modelled deterministically as fixed single-point estimate, so no 95 % confidence interval is calculated (see Table 1). ^b^Derived from *postmortem* examinations of the intestine instead of copromicroscopy. ^c^Given the lack of detailed data, it did not change over urbanization degrees. ^d^Adjusted for the rate of displayed coprophagic behaviour (see Table 2). ^e^Adjusted for the compliance of dog owners to faeces cleaning-up policies (see Table 2). ^f^Adjusted for the rate of outdoor access (see Table 2). ^g^Does not change over age groups and urbanization degrees since all stray cats and foxes release their faeces into the environment, so adjustments for outdoor access and compliance to faeces cleaning-up policies do not take place. ^h^Does not change over urbanization degrees, but only over age groups, as it was considered as a parasite-related property of a given host, irrespective of the urbanization degree where that host live

**Fig. 2:**
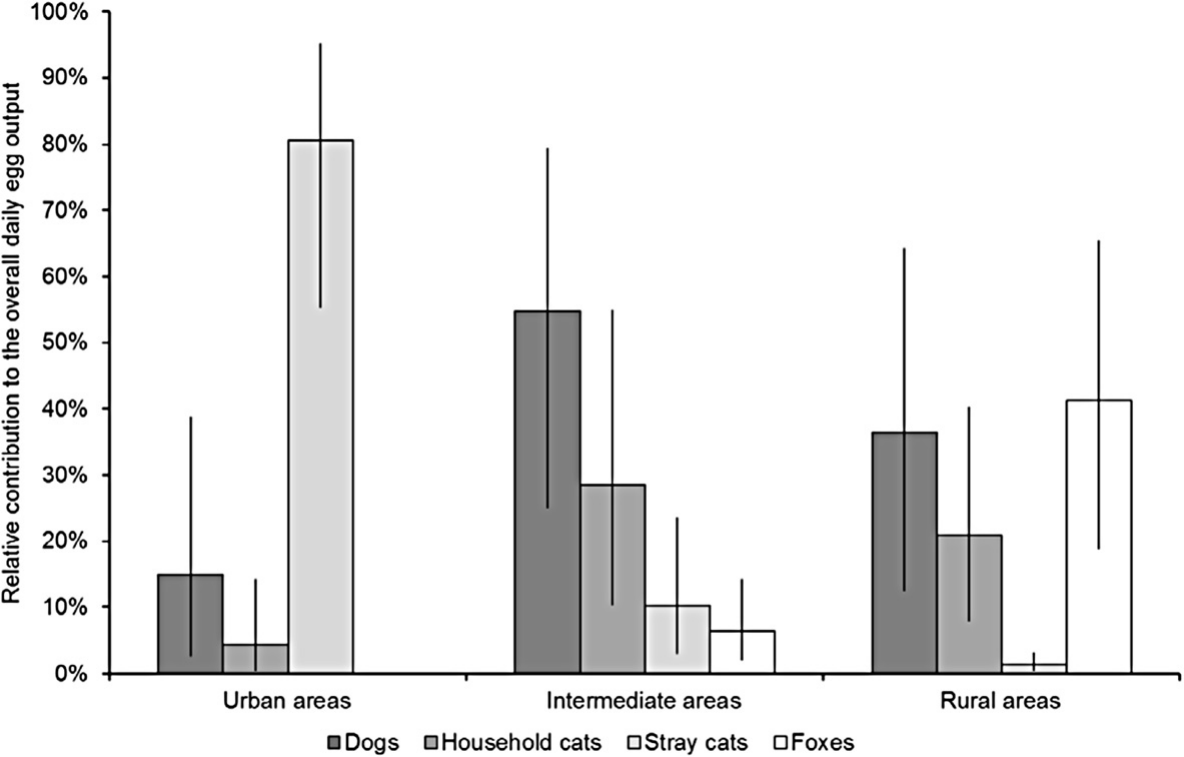
Relative contributions to environmental contamination with *Toxocara* eggs in urban, intermediate and rural areas. Estimated relative contributions (%) of non-juvenile (≥6 month-old) dogs, household cats, stray cats, and foxes to the environmental contamination with *Toxocara* eggs in urban, intermediate and rural areas in the Netherlands. Error bars represent 95 % confidence intervals

Host contributions to environmental egg contamination varied depending on the urbanization degree of the area in question (Fig. 2). In urban areas, the overall daily egg output (0.97 × 10^9^ eggs per day, corresponding to an average of 1.46 × 10^6^ eggs per km^2^ per day) was dominated by stray cats (80.7 %), followed by dogs (15.0 %), household cats (4.4 %), and foxes (<0.01 %). In intermediate areas, dogs were the main contributors (54.8 %) to the overall daily egg output (1.48 × 10^9^ eggs per day, corresponding to an average of 109,500 eggs per km^2^ per day). In rural areas, the primary contributors to the overall daily egg output (1.05 × 10^9^ eggs per day, corresponding to an average of 38,200 eggs per km^2^ per day) were foxes (41.3 %). These differences in contributions were the result of the relatively large population size of stray cats in urban areas and of foxes in rural areas, combined with a high density of dogs and household cats in intermediate areas (Table 3). Additionally, the presence of an urban-to-rural trend towards lower compliance of dog owners to dog waste clean-up policies and higher rates of outdoor access for household cats (Table 2) contributed to these differences. By contrast, foxes in urban areas and stray cats in rural areas were estimated to be few in number (Table 3), thus they appeared to contribute very little to the egg contamination in those areas.

The daily egg output of each host was dominated by adults (>12 months of age) rather than young adults (6–12 months of age). This was in spite of the generally higher prevalence and intensity of patent *Toxocara* infections in younger animals, but driven by the much higher population size of the adult host populations (Table 3). Estimated contributions of adults relative to young adults of each host were 84.2 % (95 % CI: 63.3–95.7 %) for dogs, 84.7 % (67.1–95.5 %) for household cats, 84.9 % (72.2–93.3 %) for stray cats, and 69.9 % (56.6–80.9 %) for foxes.

### Effect of deworming regimen in dogs

The resulting estimated relative contribution to the environmental contamination of non-juvenile dogs in these different scenarios is shown in Table 4. By applying a deworming frequency of twice a year (i.e. once every 6 months), scenario analysis revealed that, compared to the current deworming frequencies applied by dog owners, the estimated percent reduction in the overall daily egg output by non-juvenile dogs in the Netherlands would vary from 3.3 % (with a compliance rate of 30 %), which amounts to a 37.8 % overall contribution, to 13.8 % (with a compliance rate of 90 %), which amounts to an overall contribution of 33.7 %. With a deworming frequency of four times a year (i.e. once every 3 months), the reduction was estimated to range from 8.5 % (30 % compliance) to 29.1 % (90 % compliance), while a deworming regimen of six times a year (i.e. once every 2 months) would lead to an estimated reduction ranging from 13.8 (30 % compliance) to 44.1 % (90 % compliance). The estimated reduction of a 12 times a year deworming regimen (i.e. once every month) would vary from 28.8 (30 % compliance) to 89.6 % (90 % compliance).

**Table 4:** Estimated contribution of household dogs under different simulated deworming regimens and compliance rates

	Deworming frequency (times/year)			
	2×	4×	6×	12×
Baseline compliance	21.0 %	17.5 %	Unknown	Unknown
Baseline contribution	39.1 %	39.1 %	39.1 %	39.1 %
Simulated compliance				
30 %	37.8 (36.6–38.5)%	35.8 (32.9–37.6)%	33.7 (29.0–36.7)%	27.8 (18.0–34.4)%
50 %	36.3 (33.9–37.9)%	33.0 (27.7–36.5)%	29.7 (21.5–35.0)%	19.9 (3.3–30.9)%
70 %	35.0 (31.4–37.3)%	30.4 (22.8–35.5)%	25.7 (14.0–33.3)%	12.0 (0.0–27.5)%
90 %	33.7 (29.0–36.7)%	27.7 (17.7–34.2)%	21.9 (6.7–31.7)%	4.1 (0.0–24.3)%

The estimated percent contribution (95 % CI) of household dogs to the overall daily *Toxocara* egg output under different simulated deworming regimens and compliance rates. Baseline compliance refers to the observed compliance rates according to Nijsse et al. [9]

### Effect of dog waste clean-up policies

By increasing the observed compliance rates of dog owners on top of the reported waste clean-up policies (Table 2) by 20, 50, 70 and 90 %, the overall daily egg output of non-juvenile dogs in the Netherlands was estimated to be reduced to 32.2, 20.1, 12.0 and 4.0 % respectively (Table 5).

**Table 5:** Estimated contribution of household dogs under different compliance rates of cleaning-up faeces by owners

Compliance	Contribution to *Toxocara* egg output	
20 %		32.2 (36.4–26.7)%
50 %		20.1 (31.2–3.1)%
70 %		12.0 (26.1–0.0)%
90 %		4.0 (24.3–0.0)%

Estimated percent contribution (95 % CI) of household dogs to the overall daily *Toxocara* egg output under different simulated compliance rates of cleaning-up dog faeces

## Discussion

This study presents a quantitative approach for estimating the relative contributions of different host species, all older than 6 months of age, to the environmental contamination with *Toxocara* eggs, accounting for host density, prevalence and intensity of infection, as well as access to different areas and removal of faeces. Moreover, we assessed the effects of enforcing different deworming regimens and compliances to faeces clean-up policies for household dogs. Both published and original data were used, using the Netherlands as an example.

Even though raw meat is considered to be an important source of human *Toxocara* infections in other countries [45], infection through the ingestion of embryonated eggs from the environment is by far the most important route in the Netherlands and other Western European countries [4, 15]. Infective *Toxocara* eggs can survive for several years in the environment; therefore, effective measures to reduce human exposure to *Toxocara* should mainly aim at reducing the environmental contamination with eggs. Models like the one presented here are useful to attempt to quantify the sources of *Toxocara* eggs in a given locality as to prioritize control interventions and to assess the expected impact of such interventions. Morgan et al. [7] showed that the contributions of different hosts to the environmental contamination with *Toxocara* eggs can be quantified. Through appropriate modifications and use of additional data, our modelling framework can be extended to other regions with different urbanization degrees and different (compositions of) definitive host populations. Actual data on reported behaviors of non-juvenile dogs, cats and their owners concerning the applied deworming regimens and (compliances to) clean-up policies are included in the model. Of course leaving out the juvenile (<6-month-old) group of animals, which are unlikely to have developed age resistance, meant that the largest contributors to the environmental contamination by *Toxocara* eggs were not considered in this analysis and that emphasis was given to the larger adult host population, for which, unlike juvenile hosts, controversy exists about the need to deworm.

Our results revealed that cats contribute the most to the environmental contamination with *Toxocara* eggs by non-juvenile hosts in the Netherlands, although (household) dogs took over as the main contributors when household cats and stray cats were considered as two separate groups. This is in line with Morgan et al.’s model results [7]. However, when areas were stratified according to their degree of urbanization, host contributions appeared to differ greatly, with stray cats dominating in urban areas, dogs dominating in intermediate areas, and foxes in rural areas. The importance of cats as a putative source of *Toxocara* eggs has previously been emphasized and reported to be probably underrated [4]. Our results support the notion that controlling stray cat populations should be a priority in programmes aimed at reducing the contamination of the (urban) environment with *Toxocara* eggs. Defining the group of hosts responsible for the majority of *Toxocara* eggs shed in the environment is needed to assess the extent to which the advised *Toxocara-*control programmes may be expected to be successful in a given locality. For instance, based on our results, it seems that increasing the deworming frequency or the rate of faeces removal for non-juvenile dogs can be expected to reach the largest proportion of shedders, and also having the largest impact especially in the intermediate areas relative to urban or rural ones.

While the degree of urbanization mirrors the extent of suitable habitat for different definitive hosts, published data on the actual habitat preferences of foxes in the Netherlands are lacking. Our assumption about the distribution of the Dutch fox population over urbanization degrees was based on the urban-to-rural gradient observed in a convenience sample of shot foxes submitted by hunters for the screening for *Echinococcus multilocularis.* While it is clear that fox shooting is not usually practiced in urban areas to ensure the safety of the public, it is true that foxes have only sporadically been spotted in large Dutch cities (e.g. The Hague, Amsterdam, and Rotterdam) [46]. Therefore, most foxes appear to be dispersed over rural and intermediate areas relative to urban areas, although there may be some underestimation of the actual contribution of foxes in urban areas. For stray cats, instead, we assumed that their spatial distribution would resemble that of household cats. This meant that stray cats were found to be far more abundant in urban areas. Although it is conceivable that urban areas provide plenty of shelter and food to sustain large stray cat populations, it has been reported that stray cat dispersal might differ over seasons and different types of habitats [47, 48]. This would imply that our contribution to environmental contamination with *Toxocara* eggs of non-juvenile stray cats in urban areas might be overestimated due to insufficient insights in the spatio-temporal pattern of this cat population. Moreover, the population of stray cats in the Netherlands is actually composed of both feral (sylvatic) cats and, previously owned, abandoned stray cats which might prefer different habitats. Because key characteristics of landscape use of stray cats in the Netherlands are lacking and information about the actual dispersal of the stray cat population is scarce, outcomes of the model could not be differentiated further. However, in this study, the tendency of cats to dwell in areas with high availability of food and shelter has been decisive to assume the preference for urban areas. Future studies should focus on differentiating the contributions of these feline subpopulations, including their egg shedding patterns, habitat preferences, population structure, and possible contacts with humans.

Apart from the need to acquire more specific information about each host population, several other limitations in the model can be identified. As information in literature about the mean reproductive worm burden in adult hosts is lacking, our model made use of known EPG-values as a measure of the intensity of infection [12, 34, 40]. Modelling the number of egg-producing worms present in the intestines and their fecundity in animals older than 6 months would have probably been a more biologically sound approach. We speculate that this would have probably led to a reduction in the maximum number of eggs shed by large-sized dogs as the number of adult worms per host is not expected to be linearly correlated with its bodyweight, but rather with the dose of infective eggs/larvae ingested. Given the hosts we considered here, this assumption will have the largest effect on the modelled canine egg output, as the different breeds of dogs show the largest variation in bodyweight.

As mentioned earlier, we focussed on dogs older than 6 months because younger dogs are known to be *Toxocara* egg shedders of paramount importance [7, 10, 49]. Consensus exists that in this young age group, the propagated deworming regimen [8] and proper disposal of faeces must be enforced in any case. Conversely, the rationale of recommendations to control *Toxocara* infections in adult animals is much more arguable. If <6-month-old animals were included in the model, their contribution would have probably surpassed that of non-juvenile hosts, while the deworming advice for this age group would in fact remain the same.

The scenario analysis revealed that only in the case of a high compliance rate to a high deworming frequency (i.e. ≥50 % of owners deworming their dogs 12 times a year), the contribution of non-juvenile household dogs could be expected to be halved. It is unclear what rate of voluntary compliance to a given deworming regimen would be feasible to reach in the Netherlands or in any other country. Several studies in the Netherlands have reported a compliance of circa 40 % for deworming at least twice a year, but this was observed after conducting a campaign propagating deworming via the media or by asking clients visiting a veterinary clinic [15, 50]. Customized advice for dogs frequently shedding eggs or dogs at high risk of shedding might be more efficient in reducing the contribution of non-juvenile household dogs to the environmental contamination [9]. Blind treatments at different frequencies do not appear to be as successful as may be expected [9, 13, 51]. Considering that only about 5 % of non-juvenile household dogs actually are shedding *Toxocara* eggs at a given moment in time [9, 14, 15, 52], the question is legitimate whether it is worthwhile to invest in a policy of frequent blind treatments. The same can be said for the clean-up of dog faeces, though enforcement of mandatory removal of dog faces is perhaps more realistic, and our model showed that this would lead to results comparable to those that can be obtained with frequent deworming. Additional benefits (esthetical and hygienic) of the removal of dog faeces from the environment can play a decisive role in defining the priority of interventions. Both deworming and faeces removal were simulated separately, but the outcome of simulations assessing interaction effects between the different policies and compliances might differ from those assessing these effects independently of one another. It is therefore recommended that future studies assess these interactions and collect more information about incentives for dog owners to comply to one and/or to another policy. In addition, it is worth mentioning that we assumed an overall efficacy of 100 % for the deworming intervention, but this might not always be the case under field circumstances. Together, these results would make the (mandatory) clean-up of faeces a more pursuable *Toxocara*-control option than deworming *per se*.

Finally, because of the different defecation behaviors of household dogs, household cats, stray cats, and foxes, and the likely differences in the longevity of *Toxocara* eggs in the environment associated with these behaviors, our results might not entirely reflect the origin of the eggs actually present in the environment. Our model, therefore, was only able to predict the relative contributions of different hosts to the total number of eggs released into the environment, but not to the chance of their recovery some time afterwards.

In conclusion, a quantitative model is presented with which the relative contributions of different host species to the environmental contamination with *Toxocara* eggs can be estimated. This model expands on the previously published model of Morgan et al. [7]. Filling in gaps in current knowledge will improve the quality of data gathered to inform the model, providing more precise evidence about the most promising targets and strategies to reduce the environmental contamination with *Toxocara* eggs.

## Consent to publish

All the dog and cat owners voluntarily participated in this study and agreed on publication of the anonymised data.
